# Reducing the risk of infection: hand washing technique

**Published:** 2008-03

**Authors:** Sue Stevens

**Affiliations:** Nurse Advisor to the *Community Eye Health Journal*, International Centre for Eye Health, London School of Hygiene and Tropical Medicine, Keppel Street, London WC1E 7HT, UK.

**Figure:**
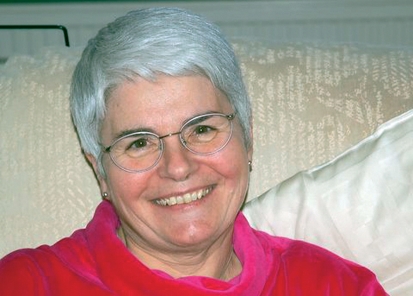


**Figure:**
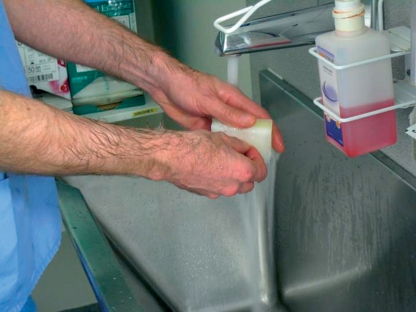
**Hand washing is a fundamental principle of infection control**

Eyes are susceptible to infection by many organisms, including gram-negative bacilli, adenoviruses, the herpes simplex virus, and fungi. Infection puts eyes at higher risk of complications after cataract surgery.

**Figure:**
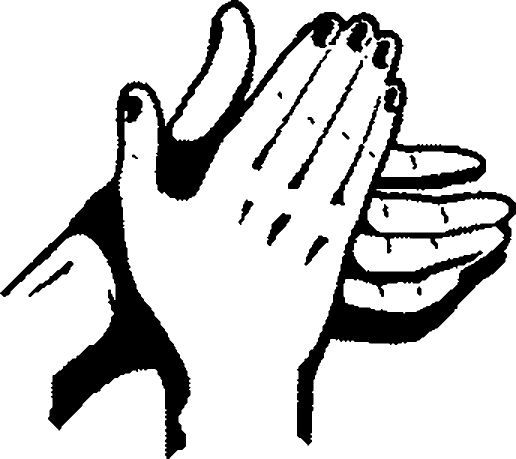
**Rub palm to palm**

Hand washing is the most important, fundamental principle of infection control. It must be strongly encouraged and practised by all disciplines in the health care setting.

Hand washing is required in the following situations:

**Figure:**
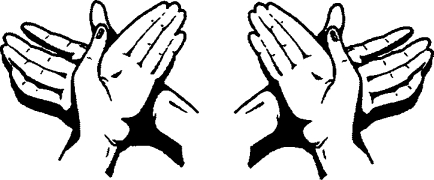
**Rub back of left hand over right palm and vice versa**

- before any aseptic procedure
- before and after handling any patient
- after handling any soiled item
- before and after handling food
- whenever hands are, or even feel, soiled
- when entering and leaving a clinical area
- after using the toilet or assisting a patient in the toilet

**Figure:**
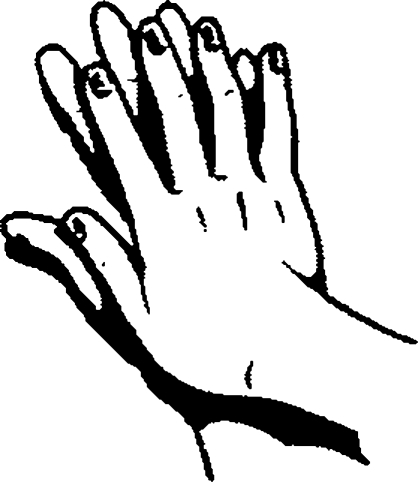
**Rub palm to palm with fingers interlaced**

Many health care workers are unaware of the need for frequent hand washing and that a certain technique is required for hand washing to be effective.

**Figure:**
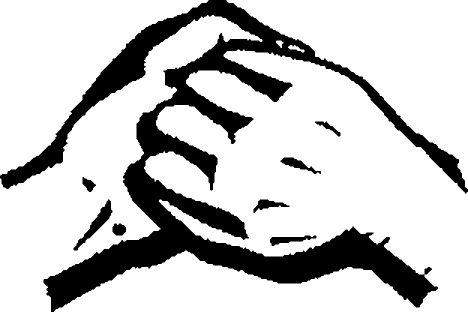
**Rub backs of fingers on opposing palms with fingers interlocked**

Written instructions for hand washing, as given below, should be displayed in all clinical areas.

- Wet hands with clean, preferably running, water
- Apply soap or cleanser
- Rub palm to palm
- Rub back of left hand over right palm
- Rub back of right hand over left palm
- Rub palm to palm with fingers interlaced
- Rub backs of fingers on opposing palms with fingers interlocked
- Rub around right thumb with left palm
- Rub around left thumb with right palm
- Rub palm of left hand with fingers of right hand
- Rub palm of right hand with fingers of left hand
- Rinse off soap with clean, preferably running, water, and dry well.

**Figure:**
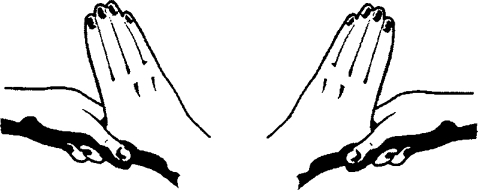
**Rub around right thumb with left palm and vice versa**

**Figure:**
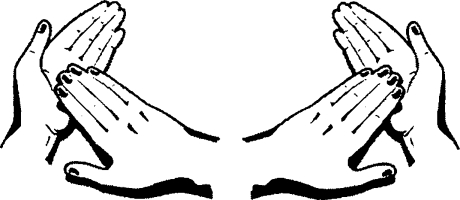
**Rub palm of left hand with fingers of right hand and vice versa**

